# Unique and redundant functions of cytoplasmic actins and nonmuscle myosin II isoforms at epithelial junctions

**DOI:** 10.1111/nyas.14808

**Published:** 2022-06-07

**Authors:** Andrei I. Ivanov, Susana Lechuga, Armando Marino‐Melendez, Nayden G. Naydenov

**Affiliations:** ^1^ Department of Inflammation and Immunity, Lerner Research Institute Cleveland Clinic Cleveland Ohio USA

**Keywords:** actin, adherens junctions, epithelial barriers, nonmuscle myosin II, tight junctions

## Abstract

The integrity and functions of epithelial barriers depend on the formation of adherens junctions (AJs) and tight junctions (TJs). A characteristic feature of AJs and TJs is their association with the cortical cytoskeleton composed of actin filaments and nonmuscle myosin II (NM‐II) motors. Mechanical forces generated by the actomyosin cytoskeleton are essential for junctional assembly, stability, and remodeling. Epithelial cells express two different actin proteins and three NM‐II isoforms, all known to be associated with AJs and TJs. Despite their structural similarity, different actin and NM‐II isoforms have distinct biochemical properties, cellular distribution, and functions. The diversity of epithelial actins and myosin motors could be essential for the regulation of different steps of junctional formation, maturation, and disassembly. This review focuses on the roles of actin and NM‐II isoforms in controlling the integrity and barrier properties of various epithelia. We discuss the effects of the depletion of individual actin isoforms and NM‐II motors on the assembly and barrier function of AJs and TJs in model epithelial monolayers in vitro. We also describe the functional consequences of either total or tissue‐specific gene knockout of different actins and NM‐II motors, with a focus on the development and integrity of different epithelia in vivo.

## Graphical abstract

The integrity and functions of epithelial barriers is regulated by the cross‐talk between intercellular junctions and the cortical actomyosin cytoskeleton. The junction‐associated cytoskeleton is built by different actin and non‐muscle myosin II isoforms. Here we overview the interactions of different cytoplasmic actins and myosin II motors with epithelial adherens and tight junctions and functional roles of these cytoskeletal proteins in regulating epithelial barrier integrity and remodeling.

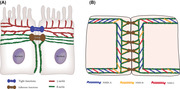

## INTRODUCTION

The establishment and function of epithelial barriers critically depend on the crosstalk between intercellular adhesions and intracellular actomyosin cytoskeleton. Intercellular adhesions that hold cells together and form the paracellular barrier are mediated by specialized epithelial junctional complexes, such as tight junctions (TJs), adherens junctions (AJs), and desmosomes.^1^, ^2^, ^3^, ^4^, ^5^ TJs and AJs are the major structural determinants of the paracellular barrier in simple epithelia. These junctions represent multiprotein plasma membrane complexes that include adhesive transmembrane and scaffolding cytosolic proteins.^1^, ^2^, ^3^, ^5^ E‐cadherin is a key transmembrane constituent of AJs that has several cytoplasmic binding partners, most notably α‐catenin, β‐catenin, and p120 catenin.^2^, ^5^ The transmembrane module of TJs is more diverse and includes members of the claudin protein family, occludin, junctional adhesion molecule‐A, and other less characterized molecules.^1^, ^3^ Members of a zonula occludens proteins family are the best‐known cytoplasmic scaffolds at TJs.^1^, ^3^ Via their cytoplasmic scaffolds, TJs and AJs are coupled to the elaborate actin cytoskeleton that includes a circumferential actin belt connected to mature AJs,^6^, ^7^ along with a more apical TJ‐associated actin filament network.^8^, ^9^ Assembly and dynamics of junction‐associated actin filaments are regulated by nonmuscle myosin II (NM‐II) motors that either slide parallel actin filaments or laterally cross‐link them into tightly packed actomyosin bundles.^10^, ^11^, ^12^, ^13^ One of the major functions of the cortical actin cytoskeleton is to create directional mechanical forces that act upon actomyosin‐coupled epithelial junctions.^12^, ^14^, ^15^ Such cytoskeletal forces are essential for initial assembly, expansion, stabilization, and disruption of epithelial AJs and TJs.^14^, ^16^, ^17^

The junction‐associated actomyosin filaments display significant structural heterogeneity at different stages of AJ and TJ biogenesis. An example is the transition from perpendicular F‐actin bundles associated with nascent dot‐like AJs into the parallel contractile actomyosin cables linked to mature AJs and TJs.^7^, ^18^, ^19^ Furthermore, stimuli‐induced junctional disassembly is accompanied by a transformation of the circumferential actomyosin belt into radial retraction fibers.^20^ Even mature epithelial junctions are known to be associated with distinct actin filament networks characterized by different dynamics.^21^, ^22^ Such structural heterogeneity of junction‐associated cytoskeletal structures is likely important for fine‐tuned regulation of different adhesive complexes and membrane domains. However, the mechanisms responsible for the assembly of diverse perijunctional actomyosin networks remain elusive.

Some studies suggest that such heterogeneity is generated by different sets of actin‐binding proteins.^23^, ^24^, ^25^ Alternatively, distinct cytoskeletal structures could also be created by biochemically different actin filaments associated with different NM‐II motors. Mammalian epithelial cells express two different actin isoforms and three NM‐II paralogs.^10^, ^13^, ^26^, ^27^, ^28^, ^29^ These isoforms can build spatially distinct populations of actomyosin filaments with different cellular functions. Relatively, little is known about how different actin isoforms and NM‐II motors regulate the integrity and functions of epithelial barriers. In this review, we briefly summarize the biochemical properties of epithelial actins and NM‐II isoforms, and discuss their involvement in assembly and remodeling of TJs and AJs in vitro, as well as in the regulation of epithelial barriers and tissue morphogenesis in vivo. We will narrow our focus on simple epithelia, where junctional association and functions of different actin and NM‐II isoforms were primarily investigated, and will not discuss less studied stratified epithelia, such as skin epidermis.

## STRUCTURE AND FUNCTIONAL ROLES OF CYTOPLASMIC ACTIN ISOFORMS

Mammals express six actin proteins each encoded by different genes.^30^ Although the most diverse array of actin isoforms is present in muscle cells, epithelial cells express two different actins called β‐cytoplasmic actin and γ‐cytoplasmic actin (referred to herein as β‐actin and γ‐actin).^27^, ^28^ While β‐actin appears to be the more abundant isoform, many epithelial tissues also express a large amount of γ‐actin protein.^27^ The two are nearly identical in sequence and differ in just four amino acid residues in their N‐terminus.^27^, ^31^ Despite their similarity, a large body of literature documents different localization of β‐actin and γ‐actin in various cells and tissues and their nonredundant functions in fundamental cellular processes, such as cell migration and cytokinesis.^26^, ^27^, ^28^, ^31^, ^32^

Several mechanisms have been proposed to explain how very subtle differences in the primary structure could result in the spatial segregation and functional diversity of cytoplasmic actins. One mechanism is based on the biochemical peculiarities of β‐actin and γ‐actin that have different kinetics of polymerization and nucleotide exchange rate in vitro.^33^ However, such biochemical differences do not prevent the copolymerization of β‐actin and γ‐actin in a cell‐free system and could be insufficient to segregate these cytoplasmic actins into different cytoskeletal structures in live cells. Another mechanism implies the isoform‐specific posttranslational modification of actins. For example, N‐terminal arginylation of β‐actin was found to be essential for proper cytoskeletal assembly and β‐actin localization at the migrating edge in motile cells.^34^ Interestingly, only arginylated β‐actin is stable in living cells, whereas arginylated γ‐actin is subjected to rapid proteasomal degradation.^35^ However, the reported level of arginylated β‐actin in various cells and tissues is very low (∼1% of total β‐actin^27^), and recent mass spectroscopy analyses failed to detect this type of actin modification under different experimental conditions.^36^ Therefore, it remains unclear if the isoform‐specific posttranslational modification could be responsible for the unique cellular distribution and functions of β‐actin and γ‐actin. A more likely mechanism could involve the interactions of cytoplasmic actins with distinct sets of actin‐binding proteins. Thus, β‐actin is known to selectively interact with important regulators of actin filament polymerization, such as diaphanous‐related formin 3 and beta‐cap73.^37^, ^38^ In contrast, γ‐actin specifically binds the Arp2/3 complex and the key actin filament depolymerizing protein, cofilin‐1.^39^ Both Arp2/3 and formin‐dependent actin polymerization play important roles in the assembly and remodeling of epithelial junctions.^40^, ^41^ However, it is currently unknown whether these different polymerization mechanisms can produce cellular actin filaments enriched in either β‐actin or γ‐actin. This is an area of interest for future studies. Finally, spatial segregation and functional specialization of cytoplasmic actins can be achieved due to the unique features of their noncoding regions. For example, β‐actin has a so‐called zip code sequence in its 3′ UTR, which is essential for targeting and local translation of β‐actin mRNA at peripheral cellular compartments, such as lamellipodia of migrating cells.^42^, ^43^ The “zip code” sequence is absent in γ‐actin 3′ UTR, which explains its lack of peripheral accumulation.^44^

## UNIQUE ROLES OF CYTOPLASMIC ACTINS IN REGULATING EPITHELIAL APICAL JUNCTIONS IN VITRO

While a significant number of published studies have addressed the unique roles of β‐actin and γ‐actin in controlling migration and division of mesenchymal and epithelial cells^37^, ^39^, ^43^, ^45^, ^46^, ^47^, ^48^, ^49^ much less is known about their involvement in the regulation of epithelial junctions and tissue barriers (Table 1).

**TABLE 1:** Effects of cytoplasmic actins depletion on structure and function of epithelial junctions in vitro and *in vivo*

Actin isoform	Depletion method	Experimental system	Effects on epithelial junctions	References
β‐Actin	siRNA‐mediated gene knockdown	SK‐CO15 human colonic epithelial cells	Increased paracellular permeability; impaired AJ, but not TJ structure; disrupted apicobasal cell polarity; attenuated AJ and TJ reassembly	50
β‐Actin	Overexpression of the Δ3’UTR mutant	MDCK cells	Attenuated AJ reassembly; increased paracellular permeability	51, 52
γ‐Actin	siRNA‐mediated gene knockdown	SK‐CO15 human colonic epithelial cells	Increased paracellular permeability; impaired TJ but not AJ structure; attenuated AJ and TJ reassembly	50
β‐Actin	Constitutive targeted gene knockout in the intestinal epithelium	C57/Bl6 mice	Increased intestinal permeability; exaggerated DSS colitis; accelerated TNFα‐induced cell death in intestinal organoids	53

Only one published study compares the roles of β‐actin and γ‐actin in the assembly and functions of TJs and AJs in SK‐CO15 human intestinal epithelial cells.^50^ This study demonstrates a selective coupling of cytoplasmic actins with different junctional complexes. Thus, in well‐polarized epithelial monolayers, β‐actin and γ‐actin was found to be specifically associated with AJs and TJs, respectively. Remarkably, TJ‐associated γ‐actin bundles appeared to have much slower turnover compared to AJ‐associated β‐actin filaments,^50^ which is consistent with the less dynamic behavior of recombinant γ‐actin polymers observed in the cell‐free system.^33^ The two cytoplasmic actins not only selectively associate with epithelial AJs and TJs, but also have unique roles in regulating these junctional complexes.^50^ Specifically, β‐actin selectively supports AJ integrity without affecting TJ structure, whereas γ‐actin controls TJ architecture without affecting AJ organization.^50^ Importantly, the depletion of either β‐actin or γ‐actin increased epithelial permeability to small ions and large uncharged molecules, which is in line with existing evidence about the cooperation of AJs and TJs in controlling the integrity of epithelial barriers.^54^

It is possible that cytoplasmic actins can regulate AJ/TJ assembly and epithelial barrier formation indirectly, given the known functions of these proteins in controlling cell migration and proliferation.^31^, ^32^ Therefore, a set of experiments involving a calcium switch was designed to test whether AJ and TJ defects observed in β‐actin or γ‐actin‐depleted epithelial cell monolayers reflect the specific effects of the cytoplasmic actins loss on epithelial junctions.^50^ In the calcium switch model, withdrawal of calcium from cell culture medium causes disassembly of all epithelial junctions, whereas readdition of calcium triggers rapid TJ and AJ reassembly.^55^ The β‐actin and γ‐actin‐depleted epithelial cell monolayers were subjected to this calcium switch to induce rapid orchestrated junctional reassembly. Notably, the depletion of either cytoplasmic actin attenuated reformation of both TJs and AJs and restoration of the perijunctional actomyosin belt.^50^ The described experiment indicates that robust remodeling of the cortical actin cytoskeleton that drives junctional reassembly requires cooperation between β‐actin and γ‐actin.

Subsequent studies in MDCK renal epithelial cells observed accumulation and local translation of β‐actin at E‐cadherin–based AJs.^51^, ^52^ Deletion of the zip code containing 3′ UTR of β‐actin attenuated its junctional localization, disrupted AJ assembly, and increased permeability of epithelial monolayers.^51^, ^52^ These results highlight the importance of the local production of actin molecules that could reinforce the junction‐associated cytoskeletal structure at the plasma membrane.

Overall, these initial studies yield exciting evidence that structurally similar cytoplasmic actins play unique functional roles at epithelial apical junctions in vitro. β‐Actin and γ‐actin cooperate during the high‐scale remodeling of the cortical actin cytoskeleton that drives the *de novo* assembly of AJs and TJs. In the stationary epithelial monolayers with a more stable actin cytoskeleton, these cytoplasmic actins have specific functions in controlling the integrity and barrier properties of TJs and AJs. It is unclear which mechanism determines such functional specialization of β‐actin or γ‐actin at epithelial junctions. The mechanism may involve interactions with different actin‐binding proteins that are selectively recruited to the cytoplasmic face of TJs and AJs.

## ROLES OF β‐ACTIN IN REGULATING INTESTINAL EPITHELIAL BARRIER AND MUCOSAL INFLAMMATION *IN VIVO*

In addition to studies addressing the cellular roles of β‐actin and γ‐actin in different in vitro systems, several published reports describe the *in vivo* functions of cytoplasmic actins using knockout and transgenic mice models.^31^ Animals with total homozygous deletion of β‐actin died at the embryonic stage due to uncharacterized developmental defects.^45^, ^56^ This signifies β‐actin as an essential regulator of mammalian survival and development. Interestingly, the embryonic lethality of β‐actin null mice can be rescued by gene editing that places a coding γ‐actin sequence into the β‐actin locus.^57^, ^58^ This indicates that the essential *in vivo* functions of β‐actin depend on its nucleotide, not amino‐acid sequence. Such coding sequence‐dependent functional differences of cytoplasmic actins were linked to the dramatic difference in their translation elongated rate that affects local actin filament assembly and remodeling.^43^ In contrast to β‐actin null mice, animals with a total knockout of γ‐actin were viable, but characterized by developmental abnormities that include attenuated growth, hearing loss, and delayed cardiac development.^59^, ^60^

The role of β‐actin in regulating gut barrier integrity and function *in vivo* was recently studied in a mouse strain with a constitutive selective knockout of this isoform in the intestinal epithelium^53^ (Table 1). These β‐actin knockout mice demonstrated increased permeability of the gut barrier with no obvious gastrointestinal abnormalities. Furthermore, the increased intestinal permeability of the knockout mice was not due to gross alteration of TJ or AJ structure or abnormal organization of epithelial actin cytoskeleton.^53^ This could be explained by the compensatory upregulation of γ‐actin expression and profound accumulation of γ‐actin at the apical junctions in the colonic epithelium of β‐actin null mice. The preserved junctional morphology and cytoskeletal architecture in β‐actin‐deficient intestinal epithelium *in vivo* contradict previous findings that loss of this actin isoform has disruptive effects on AJ structure and apicobasal cell polarity in cultured colonic epithelial cell monolayers.^50^ However, the depletion of different molecular components of the actin cytoskeleton is known to have much milder effects on intestinal epithelial homeostasis and gut barrier permeability in mice compared to their effects in model intestinal cell monolayers in vitro.^61^ These differing responses could be explained by the different levels of mechanical stress applied to epithelial junctions in vitro and *in vivo*. Thus, model epithelial monolayers cultured on stiff substrates, such as glass coverslips or plastic, are adapted to high tensile forces, whereas the intestinal epithelial barrier exists in a much softer tissue environment *in vivo* with weaker mechanical forces applied to epithelial junctions. Since high mechanical forces transduced by the actin cytoskeleton play important roles in the assembly and permeability of apical junctions,^14^, ^15^, ^16^, ^17^ even modest perturbation of actin cytoskeletal tension and contractility in vitro can lead to substantial alterations in junctional architecture. Yet, in the less mechanically challenging environment of the intestinal mucosa *in vivo*, altered organization of the force‐transducing cortical actin cytoskeleton may have less prominent effects on the organization of epithelial junctions.

Studies of β‐actin gene conditional knockout in mice strongly suggest that intestinal epithelial expression of this cytoplasmic actin plays a protective role during mucosal inflammation *in vivo*. Indeed, β‐actin gene conditional knockout mice showed more severe clinical symptoms during dextran sodium sulfate (DSS)‐induced colitis, which was associated with higher expression of inflammatory cytokines, increased accumulation of macrophages, and accelerated cell death in the colonic mucosa.^53^ Furthermore, small intestinal epithelial organoids generated from β‐actin knockout mice were more sensitive to tumor necrosis factor‐induced cell death.^53^ Overall, the described study demonstrates that β‐actin plays the unique role of regulating gut barrier integrity *in vivo* and has cell‐protective functions in the intestinal mucosa during tissue injury and inflammation.

## STRUCTURE AND BIOCHEMICAL PROPERTIES OF NM‐II MOTORS

In well‐differentiated epithelia, the perijunctional F‐actin belt is associated with the organized array of minifilaments formed by a key actin motor, NM‐II.^6^, ^9^ Together with closely related skeletal, cardiac, and smooth muscle myosins, NM‐II belongs to the class II (conventional) myosins and is widely expressed in nonmuscle tissues. The functional unit of NM‐II is a hexamer consisting of two heavy chains, two essential light chains, and two regulatory myosin light chains (RMLCs).^11^, ^62^ These hexamers self‐associate by their C‐terminal tails to form bipolar myofilaments.^63^ Phosphorylation of RMLC by myosin light chain kinase (MLCK), Rho‐associated kinase (ROCK), and some other kinases is essential for NM‐II activation and myofilament assembly.^10^, ^11^ Interactions of NM‐II myofilament with actin filaments result in generating contractile forces driven by the ATPase activity of NM‐II and lead to actin filament cross‐linking, which creates thick actomyosin bundles.^12^, ^64^ Both contractile and crosslinking activities of NM‐II are likely to be important for the assembly of the perijunctional actomyosin belt. However, the contribution of these myosin activities to the regulation of epithelial junctions is not fully understood. A significant body of literature highlights the roles of either Rho/ROCK or MLCK‐driven RMLC phosphorylation in regulating NM‐II‐dependent assembly of epithelial junctions, as well as disruption of epithelial barriers during inflammation. This mechanism has been extensively reviewed elsewhere^65^, ^66^, ^67^, ^68^, ^69^, ^70^ and will not be discussed in this review. We will focus on describing the roles of different NM‐II heavy chains in the regulation of epithelial junctions and tissue barriers in vitro and *in vivo*.

Mammalian epithelial cells express three isoforms of nonmuscle myosin heavy chains, NM‐IIA, NM‐IIB, and NM‐IIC encoded by *MYH9*, *MYH10*, and *MYH14*, respectively. These NM‐II isoforms have 60–80% amino acid sequence identity and play either unique or redundant roles in regulating different cellular processes and tissue functions.^29^, ^71^, ^72^ Studies utilizing recombinant NM‐II heavy chains revealed distinct enzymatic properties and self‐assembly of NM‐IIA, NM‐IIB, and NM‐IIC in cell‐free systems.^73^, ^74^, ^75^, ^76^, ^77^, ^78^ NM‐IIA appears to be similar to other conventional myosins, acting as a low‐duty motor, which means spending most of the kinetic cycle detached from actin filaments.^76^ By contrast, NM‐IIB is a high‐duty motor with very slow kinetics of ADP release and prolonged binding to actin filaments.^77^, ^78^ NM‐IIC is also a high‐duty motor, which is similar to NM‐IIB and has a slow rate of actin filament translocation.^73^, ^74^, ^75^ NM‐IIC self‐assembles into much shorter filaments with a lower number of myosin molecules per filament as compared to NM‐IIA/IIB.^63^ As a result, NM‐IIC has a decreased ability to cross‐link actin filaments,^63^ although the functional consequences of such reduced filament cross‐linking are not well understood. Another unique feature of NM‐IIC is its preference for β‐actin over γ‐actin, whereas neither NM‐IIA nor NM‐IIB demonstrated preferential binding to either cytoplasmic actin.^79^

The described differences in the biochemical properties of NM‐II paralogs suggest that these motors have evolved to play distinct functional roles in cells. Rapid cycling NM‐IIA motor is well‐suited to mediate actin filament sliding and contractility, whereas slowly detaching from F‐actin, NM‐IIB/IIC motors are better adapted for the regulation of static tension and strain sensing. Despite their kinetic differences, NM‐IIA and NM‐IIB are shown to cooperate in live cells by coassembling into heterodimers and heteropolymers.^80^, ^81^, ^82^ NM‐II heterooligomerization occurs at the early stages of actomyosin assembly and in the later stages, NM‐IIA and NM‐IIB can be segregated into distinct filaments based on differences in their dynamics of F‐actin binding.^81^ Thus, due to its low‐duty ratio, NM‐IIA rapidly dissociates from maturing actin filaments, whereas the high‐duty ratio NM‐IIB remains attached to these filaments. Such self‐sorting processes produce a diverse array of actomyosin structures with a gradient of NM‐IIA and NM‐IIB subunits determining their different contractility, intracellular localization, and functions.^29^

## EPITHELIAL EXPRESSION AND LOCALIZATION OF DIFFERENT NM‐II ISOFORMS

Since NM‐II isoforms could cooperate or compete for actin filament binding, their involvement in the regulation of different cellular processes should be dependent on the abundance and localization of these cytoskeletal motors. While all three NM‐II heavy chains have been detected in different epithelial cells, NM‐IIA appears to be predominantly expressed. Mass spectroscopic analysis revealed the highest proportion of NM‐IIA, ranging from 54% to 96% of all NM‐II heavy chains, in cultured intestinal, mammary, renal, and cervical epithelial cells.^83^, ^84^, ^85^ Expression of NM‐IIB and NM‐IIC is highly variable among different epithelial cell lines, with reported relative expression of NM‐IIB and NM‐IIC in the range of 0–20% and 3–45% of all NM‐II motors, respectively.^83^, ^84^, ^85^ It is noteworthy that some well‐differentiated human epithelial cell lines, such as T84 colonic and HPAF‐II pancreatic epithelial cells, do not express detectable NM‐IIB protein^83^, ^86^ (A.I. Ivanov, personal communication). Furthermore, an interesting NM‐II isoform switch phenomenon was observed during the epithelial‐to‐mesenchymal transition of normal NMuMG mammary gland epithelial cells.^87^ Treatment of these cells with transforming growth factor‐β resulted in their conversion from epithelial to spindle‐like mesenchymal morphology, which was accompanied by a dramatic upregulation of NM‐IIB expression and decreased NM‐IIC protein and mRNA levels.^87^ These results suggest that NM‐IIA and NM‐IIC expression could be a general feature of differentiated epithelial cells, whereas NM‐IIB could be expressed in specialized epithelial cells or induced during epithelial dedifferentiation and transformation. In several well‐differentiated epithelial cell lines, all three NM‐II isoforms are shown to be enriched at the circumferential F‐actin belt and produce very similar junctional labeling patterns.^85^, ^86^ Furthermore, super‐resolution microscopy revealed an assembly of NM‐IIA, NM‐IIB, and NM‐IIC into supramolecular sarcomeric‐like arrays within the perijunctional F‐actin belt,^6^, ^88^ highlighting this belt as the contractile force‐generating structure.

Epithelial expression of different NM‐II isoforms has also been studied in mouse tissues. The results are consistent with the in vitro data that indicate NM‐IIA is an obligate myosin motor that can be coexpressed with NM‐IIC or NM‐IIB in a tissue‐specific fashion. Thus, in mouse small and large intestine, NM‐IIA and NM‐IIC are abundant in the epithelial layer, whereas NM‐IIB is abundant in the subepithelial compartment but undetectable in the colonic epithelium as seen by immunofluorescence labeling.^89^, ^90^ Likewise, NM‐IIA and NM‐IIC are highly expressed in airway epithelial cells in developing lungs, whereas NM‐IIB expression is prominent in mesenchymal cells and low in airway epithelium.^91^ In the murine squamous epithelium of the tongue, only NM‐IIA and NM‐IIC are expressed, while NM‐IIB protein is absent according to immunolabeling and mass spectroscopic analysis.^92^ By contrast, NM‐IIA and NM‐IIB are ubiquitously expressed in kidneys, including the ureteric bud and nephric duct‐derived epithelia.^93^, ^94^ In different mouse tissues, both NM‐IIA and NM‐IIC are enriched at intercellular contacts and at the apical pole of epithelial cells, where they colocalize with TJ and AJ proteins.^89^, ^90^, ^91^, ^92^

## DISRUPTION OF EPITHELIAL JUNCTIONS BY PHARMACOLOGICAL INHIBITION OF NM‐II MOTORS IN VITRO

Initial studies examining the roles of NM‐II motors in the regulation of epithelial junctions were carried out using a specific chemical inhibitor of myosin II called blebbistatin.^95^ Blebbstatin markedly slows down the ATPase activity of NM‐II and keeps it in the actin‐detached state.^96^ Studies that utilized blebbistatin demonstrated multiple roles of NM‐II in regulating either steady‐state integrity of epithelial junctions or junctional remodeling (disassembly and reassembly; Table 2).

**TABLE 2:** Effects of the pharmacological NM‐II inhibitor, blebbistatin, on AJ and TJ structure and remodeling in vitro

Cell line	Experimental conditions	Effects on epithelial junctions	References
Eph4 mammary epithelial cells	Confluent cell monolayers	No changes in TEER and fluorescein flux; no changes in the TJ structure	97
MDCK kidney epithelial cells	Confluent cell monolayers	Increased TEER; no changes in FITC dextran flux and TJ structure	98
T84 colonic epithelial cells	Confluent cell monolayers	Slightly decreased TEER	20
NRK kidney epithelial cells	Steady‐state monolayers	Disassembled punctate AJs and TJs	99
MCF‐7 mammary epithelial cells	Steady‐state monolayers	Disrupted steady‐state AJs	100
T84 cells	Calcium switch	Attenuated TJ reassembly; no effect on AJ reassembly	18
MDCK cells	Calcium switch	Prevented TJ reassembly	101
T84 cells	Calcium depletion	Attenuated AJ and TJ disassembly	20
T84 cells	Interferon‐γ treatment	Attenuated TJ disassembly	102
HPAF‐II pancreatic epithelial cells	Octylindolactam‐V treatment	Attenuated AJ and TJ disassembly	103
Kidney tubular epithelial cells	Angiotensin II treatment	Attenuated AJ disassembly	104

In moderately differentiated MCF‐7 human mammary epithelial cells and normal rat kidney epithelial cells, blebbistatin treatment caused the rapid displacement of E‐cadherin, from cell–cell contacts and reduced perijunctional F‐actin cables associated with cadherin‐based AJs.^99^, ^100^ Furthermore, in N‐cadherin‐expressing myogenic cells plated on N‐cadherin‐coated micropillar substrates, blebbistatin treatment resulted in a 50% decrease of contractile forces in parallel to impaired formation of adhesive cadherin clusters and associated actin filaments.^105^ In contrast to the detrimental effects of blebbistatin on cadherin‐based AJs in poorly differentiated epithelial cells, pharmacological inhibition of NM‐II failed to disrupt tight barriers in well‐differentiated epithelial monolayers (Table 2). Thus, blebbistatin did not have consistent effects on transepithelial electrical resistance (TEER) in confluent colonic, renal, and mammary epithelial monolayers.^20^, ^97^, ^98^ Furthermore, blebbistatin did not affect the steady‐state TJ integrity of these cells.^97^, ^98^ The data suggest different requirements for NM‐II activity in controlling the structure and function of immature and mature epithelial junctions.

In contrast to its variable effects on the organization of steady‐state epithelial junctions, pharmacological inhibition of NM‐II motor was shown to consistently suppress stimuli‐induced remodeling of TJs and AJs (Table 2). For example, blebbistatin treatment selectively inhibited calcium‐stimulated TJ reassembly without affecting the reestablishment of AJs.^18^, ^99^, ^101^ Interestingly, the attenuation of TJ reassembly in NM‐II‐inhibited epithelial cells was linked to the impaired establishment of apicobasal cell polarity, where an apical plasma membrane marker, syntaxin 3, and the TJ protein, occludin, were locked in the intermediate vacuolar‐like structures at the lateral cell–cell contacts.^18^ Furthermore, inhibition of NM‐II motors attenuated the opposite process: stimuli‐induced disruption of epithelial junctions in various experimental systems. Specifically, blebbistatin treatment attenuated TJ and AJ disassembly in calcium‐depleted human colonic epithelial monolayers,^20^ and in pancreatic ductal cells treated with the tumor promoter, octylindolactam‐V.^103^ Likewise, blebbistatin prevented the disruption of E‐cadherin‐based adhesions in renal tubular epithelial cells challenged with angiotensin II^104^ and attenuated a selective TJ disassembly in colonic epithelial cells exposed to interferon‐γ.^102^ The studies implicating NM‐II contractility in stimuli‐induced disassembly of epithelial junctions are consistent with known roles of NM‐II activating protein kinases MLCK and ROCK in the disruption of epithelial barriers under inflammatory conditions.^65^, ^66^, ^67^, ^68^, ^69^, ^70^

## ROLES OF DIFFERENT NM‐II MOTORS IN REGULATING TJ AND AJ INTEGRITY AND DYNAMICS IN VITRO

The association of all three NM‐II motors with the perijunctional F‐actin belt^6^, ^85^, ^86^ suggests that different NM‐II isoforms cooperate in controlling the integrity and dynamics of epithelial TJs and AJs. Several studies have revealed the elaborate functional crosstalk between NM‐IIA and NM‐IIB at epithelial junctions in vitro. The first study addressing the roles of different NM‐II isoforms in regulating junctional structure and remodeling was carried out in SK‐CO15 human colonic epithelial cells that express all three NM‐II motors.^86^ In this study, the selective downregulation of either NM‐IIA, IIB, or IIC expression using isoform‐specific siRNAs demonstrated a unique role of NM‐IIA in regulating different steps of junctional biogenesis. Thus, NM‐IIA depletion attenuated TEER development, indicating the delayed establishment of the epithelial barrier. When NM‐IIA‐deficient SK‐CO15 cell monolayers were subjected to the calcium switch, they displayed both defective AJ and TJ disassembly during calcium depletion and impaired reformation of these junctional complexes after restoration of extracellular calcium.^86^ The underlying mechanisms involve junction‐associated actin cytoskeleton since NM‐IIA‐depleted cells failed to assemble contractile F‐actin rings and did not efficiently restore the circumferential F‐actin belt at the calcium depletion and repletion steps, respectively. These effects appeared to be NM‐IIA specific since depletion of neither NM‐IIB nor NM‐IIC affected epithelial barrier formation and AJ/TJ remodeling.^86^

Subsequent studies examined the crosstalk between NM‐IIA and NM‐IIB in the regulation of AJ formation and stability at high temporal and spatial resolution.^85^, ^106^, ^107^ They confirmed the key roles of NM‐IIA in driving AJ assembly, strengthening homotypic E‐cadherin adhesions, and promoting the formation of other junctional complexes, such as TJs and desmosomes in mammary and renal epithelial monolayers (Table 3). Additionally, NM‐IIA is shown to regulate tensile forces at epithelial AJs.^106^, ^108^ Defects of AJ assembly in NM‐IIA‐depleted MDCK cells were rescued by the expression of the unfolded, active α‐catenin mutant that is capable of simultaneous interactions with both E‐cadherin and actin filaments.^107^ Additionally, *in situ* proximity biotinylation revealed force‐dependent interactions between α‐catenin and NM‐IIA.^109^ This suggests that NM‐IIA‐driven forces result in the activating unfolding of α‐catenin molecule, thereby promoting epithelial junction assembly and interactions with the cortical cytoskeleton.

**TABLE 3:** Effects of downregulation of different NM‐II isoforms on epithelial junctions in vitro

NM‐II isoform	Downregulation method	Cell line	Junctional phenotypes	References
NM‐IIA	siRNA‐mediated gene knockdown	SK‐CO15 cells	Attenuated TEER development. Inhibited AJ/TJ assembly and disassembly during calcium switch	86
NM‐IIA	shRNA‐mediated gene knockdown	MCF‐7 cells	Disrupted steady‐state AJ; no effect on TJ structure	85
NM‐IIA	CRISPR/Cas9 gene knockout	MDCK cells	Disrupted AJ, TJ, and desmosomes	107
NM‐IIA	shRNA‐mediated gene knockdown	MDCK cells	Attenuated AJ assembly; decreased tensile force at AJ	106
NM‐IIB	siRNA‐mediated gene knockdown	SK‐CO15 cells	No effect on TEER and TJ/AJ remodeling	86
NM‐IIB	shRNA‐mediated gene knockdown	MCF‐7 cells	No effect on AJ structure; disrupted junction‐associated F‐actin	85
NM‐IIB	shRNA‐mediated gene knockdown	MDCK cells	No effect on AJ assembly; abnormal structure of AJ‐associated F‐actin	106
NM‐IIC	siRNA‐mediated gene knockdown	SK‐CO15 cells	No effect on either TEER or AJ and TJ remodeling	86

NM‐IIA appears to not be the sole NM‐II isoform regulating epithelial junctions since the contribution of NM‐IIB to the organization of epithelial cell–cell adhesions was also reported (Table 3). While NM‐IIB depletion in MCF‐7 cells did not affect E‐cadherin accumulation at intercellular contacts, it did cause multiple breaks in the linear continuity of AJs.^85^ Consistently, the loss of NM‐IIB in MDCK cells resulted in the assembly of twisted and disoriented E‐cadherin–based junctions.^106^ These distinct effects of NM‐IIA and NM‐IIB depletion on the AJ architecture could be explained by different molecular organization of NM‐II isoforms at epithelial junctions. Super‐resolution microscopy of MDCK cell monolayers revealed an association of NM‐IIA with thick F‐actin bundles running in parallel to epithelial AJs and locating a few microns from the transmembrane E‐cadherin complexes.^106^ Similar localization of junctional NM‐IIA was reported in human endothelial cells.^110^ In contrast, NM‐IIB was associated with a juxtamembrane branched actin network and displayed colocalization with the key AJ component β‐catenin.^106^ Consistently, the knockdown of NM‐II isoforms demonstrated that NM‐IIA controls the organization of parallel contractile F‐actin bundles, while NM‐IIB organizes the juxtamembrane F‐actin meshwork connecting the perijunctional bundles to E‐cadherin complexes at the plasma membrane.^106^ Despite knowing the high expression of NM‐IIC in certain types of epithelia and its specific junctional localization, nothing is known about its roles in regulating epithelial junctions.^6^, ^86^ The only study in colonic epithelial cells did not observe gross defects in AJ/TJ organization and remodeling (disassembly and reassembly) after NM‐IIC depletion.^86^ Generally, NM‐IIC function in mammalian cells remains poorly investigated, although this actin motor has been implicated in the regulation of cytokinesis in lung cancer cells,^111^ extracellular matrix adhesion in neurons,^112^ and microvillar growth in intestinal epithelial cells.^113^ Together, the described studies demonstrate that NM‐IIA plays key roles in regulating assembly, barrier function, and remodeling of apical junctions in different model epithelia. NM‐IIB could aid NM‐IIA in regulating AJ stability by controlling assembly and spatial organization of the junction‐associated F‐actin cytoskeleton.

## FUNCTIONS OF NM‐II MOTORS IN REGULATING EPITHELIAL BARRIERS *IN VIVO*

Physiological functions of different NM‐II isoforms have been extensively studied *in vivo* by generating mouse models with tissue‐specific deletion of these cytoskeletal motors.^84^, ^114^ Several studies examined the effects of either individual or dual knockouts of NM‐II isoforms in mice on the integrity and functions of different epithelial barriers (Table 4).

Germline ablation of NM‐IIA in mice resulted in embryonic lethality before gastrulation due to the inability to form polarized visceral endoderm.^115^ NM‐IIA is the only NM‐II paralog expressed in visceral endoderm and its loss resulted in defective cell–cell adhesions due to mislocalization of E‐cadherin and β‐catenin from AJs.^115^ Interestingly, the replacement of NM‐IIA with either NM‐IIB^114^ or NM‐IIC^119^ rescued the cell–cell adhesion defects in visceral endoderm and supported animal development beyond gastrulation. These knockin mice still died at the embryonic stage due to abnormal angiogenesis and placenta formation.^114^, ^119^ The described studies have indicated isoform independent requirement of NM‐II for the formation of cell–cell adhesions in the visceral endoderm, but a unique functional role of NM‐IIA in placenta development. In contrast to NM‐IIA gene knockout, germline ablation of other NM‐II isoforms did not result in noticeable defects in epithelial barriers *in vivo*. Mice with a total knockout of NM‐IIB were characterized by embryonic or perinatal lethality due to heart failure.^120^ The heart failure was from cardiac myocyte hypertrophy and abnormal myofilament organization, although the structure of intercellular junctions in NM‐IIB–deficient cardiomyocytes remained normal.^120^ No vivid morphological or functional changes in the major epithelial‐rich organs: gastrointestinal tract, kidneys, or lungs, were reported in NM‐IIB gene knockout mice. Mice with the total knockout of the NM‐IIC gene were also created and characterized.^84^ Despite NM‐IIC being highly expressed in adult lungs and enriched at epithelial junctions, ablation of this isoform did not result in any morphological or functional abnormalities *in vivo*.^84^ Of note, NM‐IIC gene knockout mice showed increased accumulation of NM‐IIA and NM‐IIB at the perijunctional actin belt of their organ of Corti epithelium,^6^ suggesting a functional compensation for NM‐IIC loss by other myosin isoforms.

**TABLE 4:** Functional effects of NM‐IIA deletion in different epithelia *in vivo*

Type of knockout	Tissue specificity	Phenotype	References
Germline NM‐IIA knockout	Whole body	Embryonic lethality; defective cell–cell adhesions in visceral endoderm	115
Constitutive NM‐IIA knockout	Intestinal epithelium	Increased gut permeability; altered localization of AJ/TJ proteins in colonic epithelium; increased sensitivity to DSS colitis	89
Dual knockout (inducible NM‐IIA and constitutive NM‐IIC)	Intestinal epithelium (NM‐IIA) and whole body (NM‐IIC)	Impaired intestinal crypt development	90
Inducible NM‐IIA knockout	Intestinal epithelium	Impaired intestinal crypt development; increased epithelial necroptosis	116
Constitutive NM‐IIA knockout	Renal podocytes	Abnormal podocyte adhesion; glomerulosclerosis	117
Expression of NM‐IIA D1424N mutant	Whole body	Abnormal podocyte adhesion; glomerulosclerosis	117
Expression of NM‐IIA E1841K mutant	Whole body	Abnormal podocyte adhesion; glomerulosclerosis	117
Constitutive NM‐IIA knockout	Renal metanephric mesenchyme	Proximal tubule dilation and renal failure	94
Dual constitutive NM‐IIA/NM‐IIB knockout	Ureteric bud	Hydronephrosis; alterations in epithelial morphology	93
Dual inducible NM‐IIA/NM‐IIB knockout	Renal tubular epithelium	Tubular injury and renal failure	118

Subsequent studies that utilized mice models with tissue‐specific deletion of NM‐IIA revealed multiple roles of this actin motor in different murine epithelia. Constitutive deletion of the NM‐IIA gene in the intestinal epithelium increased permeability of the gut barrier and altered the molecular organization of colonic epithelial TJs and AJs manifested by mislocalization of claudin‐7 and β‐catenin proteins.^89^ The overall colonic epithelial morphology and organization of the perijunctional F‐actin cytoskeleton appeared unchanged in NM‐IIA gene conditional knockout mice.^89^ In another study, a dual deletion of NM‐IIA and NM‐IIC genes inhibited apical constriction and invagination of intestinal epithelial cells, thereby impairing normal crypt morphogenesis.^90^ This study suggests that NM‐II activity is required only at the onset of crypt invagination, whereas in the established intestinal epithelial crypts, a dual NM‐IIA/IIC gene knockout does not have marked effects on epithelial cell shape and morphology.^90^ The most recent study of tamoxifen‐induced deletion of NM‐IIA in the intestinal epithelium reported abnormal development of small intestinal crypts following NM‐IIA ablation, which was associated with depletion of Lrg5^+^ stem cells and intestinal epithelial cell necroptosis.^116^

Disruption of the gut barrier integrity in intestinal epithelial NM‐IIA gene knockout mice resulted in low‐scale spontaneous mucosal inflammation without causing major clinical symptoms of gastrointestinal disorders.^89^ Furthermore, loss of NM‐IIA in the intestinal epithelium markedly exaggerated DSS‐induced colitis, manifested by more severe epithelial erosion, infiltration of inflammatory cells, and barrier disruption.^89^ Notably, increased permeability of the gut barrier has been associated with a large variety of human gastrointestinal and systemic inflammatory disorders.^121^, ^122^, ^123^ Such barrier leakage is thought to contribute to the development of diseases by increasing bodily exposure to luminal bacteria, although very few tools exist to directly test such a hypothesis *in vivo*. Mice with an intestinal epithelial‐specific knockout of NM‐IIA that are characterized by leaky gut without pathological changes in their intestinal mucosa may serve as a valuable model to examine the contribution of gut leakage to the pathophysiology of different inflammatory disorders.

Kidneys represent another epithelium‐rich organ, where *in vivo* effects of inactivating NM‐II motors were investigated. Initial studies involved transgenic mice expressing the most common mutations of human NM‐IIA either in the globular head (R702C and D124N) or the tail (E1841K) domain of this protein,^117^ which interfere with myosin motor and cross‐linking activities, respectively. Transgenic animals expressing all NM‐IIA mutants developed kidney disease manifested by albuminuria and progressive glomerulosclerosis. A similar phenotype consisting of impaired podocyte adhesion and progressive glomerulosclerosis was observed in mice with specific deletion of the NM‐IIA gene in podocytes,^117^ thereby signifying a unique role of NM‐IIA in regulating the podocyte‐dependent glomerular barrier. Later studies of dual NM‐IIA and NM‐IIB gene conditional knockout mice examined the roles of these cytoskeletal motors in kidney development.^93^, ^94^, ^118^ Kidney morphogenesis is driven by two embryonal precursor tissues: the metanephric mesenchyme and the ureteric bud. The former precursor develops into glomeruli and renal tubules, whereas the latter gives rise to collective ducts and ureters. Ureter bud‐specific deletion of NM‐IIA did not result in any phenotype, whereas mesenchyme‐specific ablation of NM‐IIA triggered proximal tube dilatation and renal failure.^94^ This indicates differential functions of NM‐IIA in controlling the development of the lower and upper parts of the urinary tract. Dual knockout of NM‐IIA and NM‐IIB genes in either metanephric mesenchyme or ureteric bud resulted in unique phenotypes. For example, the mesenchyme‐specific NM‐IIA/NM‐IIB gene knockout caused a dramatic reduction in most of the nephron components, resulting in animal lethality shortly after birth.^94^ The nascent nephrons in dual‐knockout mice did not form a continuous lumen, most likely due to impaired apical constriction and defective epithelial cell polarity of elongating tubules. A dual NM‐IIA/NM‐IIB gene knockout in the ureteric bud obliterated the connection between the ureters and bladder causing hydroureter/hydronephrosis.^93^ These morphological abnormalities were associated with marked alterations in the structure of epithelial layers manifested by the formation of abnormal basal protrusions and apical extrusions of epithelial cells.^93^ Interestingly, E‐cadherin accumulation at AJs was reduced in ureteric bud‐specific dual NM‐IIA/NM‐IIB gene knockout mice, which could be responsible for the impaired epithelial layer integrity and cell extrusion.^93^

Functions of NM‐II motors were also investigated in the adult renal epithelium by using a mouse model with inducible dual ablation of NM‐IIA/NM‐IIB in renal tubules that does not affect the glomerular expression of these proteins.^118^ Since NM‐IIC expression in the adult renal epithelium is very low,^94^ a dual knockout of NM‐IIA and NM‐IIB genes likely eliminated the entire NM‐II activity in renal tubules. This inducible NM‐II gene knockout resulted in progressive kidney diseases from tubular injury and immune cell infiltration.^118^ While the assembly of epithelial junctions was not examined in the NM‐II–deficient renal tubes, the tubular epithelial cells retained their apicobasal polarity based on unaltered localization of apical and basolateral membrane transporters and pumps. Yet, some plasma membrane proteins appeared to be mislocalized and internalized in NM‐II‐depleted renal epithelium along with expansion of the endoplasmic reticulum and activation of the unfolded protein response.^118^

The progressive renal pathology observed in NM‐II gene knockout mice is particularly interesting in light of clinical data that link genetic variants of NM‐IIA with development of kidney diseases. Mutations of the human NM‐IIA gene can result in an autosomal‐dominant disorder known as *MYH9*‐related disease (*MYH9*‐RD).^124^, ^125^ This disease appears to be associated with more than 80 different mutations of NM‐IIA.^64^ While early and predominant pathological manifestations of *MYH9*‐RD are hematological abnormalities, such as thrombocytopenia and platelet macrocytosis, about 25% of the patients develop late‐onset kidney disease.^126^, ^127^, ^128^ The disease presents as rapidly progressing proteinuric nephropathy eventuating in the end‐stage renal failure^127^ and is characterized by podocyte injury and loss of their cell‐cell and cell–matrix contacts.^126^ Interestingly, mutations in the motor domain of NM‐IIA are predictive of the development of kidney diseases in *MYH9*‐RD patients, whereas mutations in its tail domain have a much lower risk of renal and other complications.^128^ These examples suggest the potential role of NM‐IIA mutations in the development of human diseases associated with disruption of different tissue barriers.

The described gene knockout studies allow us to make the following conclusions regarding NM‐II roles in regulating epithelial barriers and morphogenesis *in vivo*. First, NM‐IIA may either have a unique role in controlling the development of epithelial barriers and organization of apical junctions or cooperate with other NM‐II isoforms to ensure normal establishment and functions of tissue barrier. Second, loss of either NM‐IIA alone or dual depletion of NM‐IIA/IIB commonly results in tissue inflammation due to increased permeability of epithelial barriers and immune cell infiltration. Third, NM‐II activity is particularly important at the early stages of epithelial morphogenesis in different organs. Surprisingly, already developed adult epithelial tissues could tolerate almost total elimination of NM‐II without marked disruption of tissue integrity and cellular architecture *in vivo*. It is unclear how epithelial cells compensate for the loss of NM‐II functions; however, possible mechanisms could involve unconventional myosins and different F‐actin cross‐linking proteins.

## CONCLUSIONS

**FIGURE 1:**
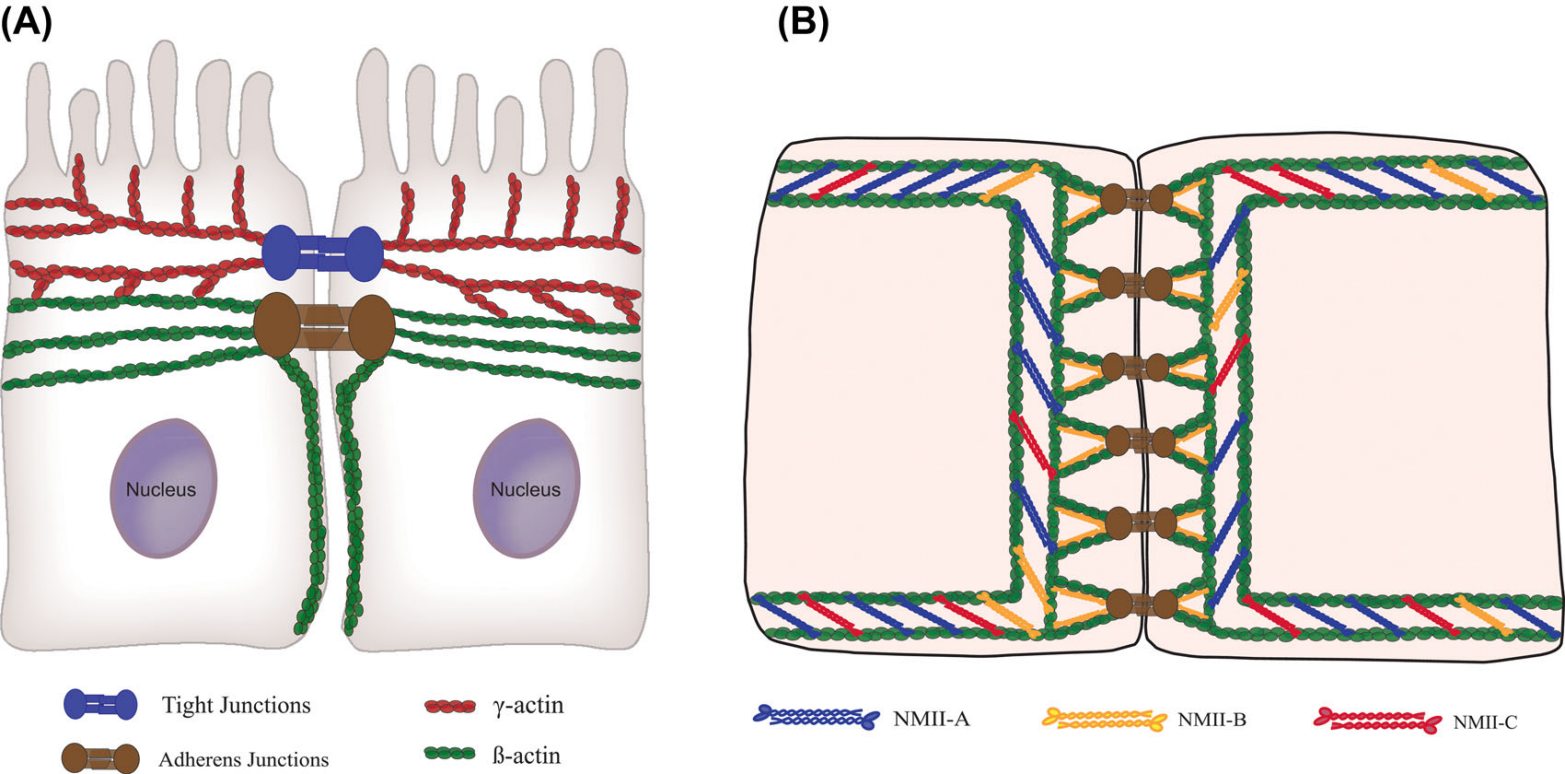
Proposed model for the association of different cytoplasmic actins and NM‐II isoforms with epithelial apical junctions. (A) In simple columnar epithelia, β‐actin‐based filaments are predominant in the perijunctional F‐actin belt coupled to AJ; they also create the cortical actin network along the lateral plasma membrane. By contrast, γ‐actin‐based filaments are associated with TJs and create the apical actin network that involves microvilli. (B) All three NM‐II motors are involved in the assembly of AJ‐associated actomyosin structures. NM‐IIA and NM‐IIC represent the most abundant NM‐II isoforms in the circumferential belt, while NM‐IIB is uniquely associated with the juxtamembrane actin network directly coupled with E‐cadherin clusters at the plasma membrane

The elaborate and dynamic network of junction‐associated actomyosin filaments regulates epithelial junctions’ establishment and remodeling. Mechanical forces generated by these cytoskeletal structures control all stages of junctional biogenesis, epithelial polarization, and tissue morphogenesis. The perijunctional cytoskeleton is created by coassembly of different cytoplasmic actins and NM‐II motors that have distinct biochemical properties and may control different steps of TJ/AJ assembly, maturation, and disintegration. The existing evidence allows us to propose an early model for the functional interplay between different actin and myosin isoforms at epithelial junctions (Figure 1). This model suggests a spatial separation and functional specialization of β‐actin and γ‐actin. Specifically, β‐actin is responsible for building F‐actin structures associated with AJs and lateral cell–cell contacts, whereas γ‐actin assembles the TJ‐coupled filaments. NM‐IIA and NM‐IIC are likely to be responsible for the assembly of the contractile circumferential actomyosin belt controlling AJ and TJ integrity (Figure 1). NM‐IIB isoform could either participate in the perijunctional belt assembly in some types of epithelia or associate with the juxtamembrane actin filament network that links the contractile F‐actin belt with AJs. The configuration of NM‐II isoforms at epithelial TJs is less clear; however, the functional studies highlight NM‐IIA as the most important TJ motor. This hypothetical model could provide a valuable framework for future studies to address many unanswered questions. So far, little is known about the mechanisms responsible for selective association of β‐actin and γ‐actin with different junctional complexes. Likewise, our understanding of cytoplasmic actins’ functional roles in regulating epithelial barriers *in vivo* remains in infancy. While a large body of evidence implicates the most abundant epithelial motor, NM‐IIA, in the regulation of epithelial barriers, the involvement of other NM‐II isoforms is poorly understood. Furthermore, relatively little is known about how different NM‐II motors control cell–cell adhesions and barrier properties of different epithelial layers *in vivo*. Finally, much should be learned about the signaling mechanisms, which control the activity and assembly of different NM‐II motors under normal homeostatic conditions and during tissue inflammation and tumorigenesis. These important questions could be addressed by adapting novel experimental models, such as primary epithelial cell organoids and animals with epithelial‐specific knockouts of different actin and NM‐II isoforms. Such studies are expected to provide critical novel insights into understanding the roles and mechanisms of cytoskeletal regulation of epithelial barriers in health and diseases.

## COMPETING INTERESTS

All authors declare no competing interests.

## AUTHOR CONTRIBUTIONS

All authors contributed to the conceptualization, writing, and editing of this review.

### PEER REVIEW

The peer review history for this article is available at: https://publons.com/publon/10.1111/nyas.14808.
